# Loss of Cyclin-Dependent Kinase Inhibitor Alters Oncolytic Adenovirus Replication and Promotes More Efficient Virus Production

**DOI:** 10.3390/cancers10060202

**Published:** 2018-06-15

**Authors:** Naseruddin Höti, Tamara Jane Johnson, Wasim H. Chowdhury, Ronald Rodriguez

**Affiliations:** 1 Department of Pathology, Johns Hopkins School of Medicine, Baltimore, MD 21205, USA; nhoti1@jhmi.edu; 2 James Brady Urological Institute, The Johns Hopkins School of Medicine, Baltimore, MD 21205, USA; tamarajane.johnson@gmail.com; 3 Department of Urology, University of Texas Health Science Center San Antonio, San Antonio, TX 78229, USA; ChowdhuryW@uthscsa.edu

**Keywords:** adenovirus replication, p21, oncolytic adenovirus

## Abstract

We elucidate the role of p21/Waf-1, a cyclin-dependent kinase inhibitor, on the oncolytic infection and replication cycle of adenovirus by studying both mRNA and adenoviral proteins expression. We found that infection in the absence of p21 causes a significant increase in adenoviral genomes and late gene expression. Similarly, the oncolytic adenoviral infected p21^−/−^ cells have earlier formation of replication foci and robust replication kinetics that were not observed in the wild type p21/Waf-1 intact cells. These findings suggest a culmination that the presence of intact p21 in host cells causes defects in the oncolytic viral life cycle which results in the production of immature and noninfectious particles.

## 1. Introduction

The utility of the adenovirus as a vector for gene therapy has been well documented and relies on the importance of E1A expression [1,2,3]. Replacing the constitutive promoter with tissue- or cancer-specific promoters upstream from E1A restricts replication to specific tissues for oncolytic virus therapy [4,5]. Although the specificity achieved through using tissue-specific promoters reduces replication in nontargeted tissues and organs thereby reducing toxicity, it also attenuates viral replication due to lower activity of selected promoters [6]. Understanding the mechanisms of adenovirus replication and the barriers that the virus must overcome may help design the next generation of gene therapy vectors.

Inhibition in adenovirus replication was observed when valproic acid treatment was combined with oncolytic adenoviruses, which was attributed to the upregulation of p21/Waf-1(p21) expression [7]. p21 is a member of the cip/kip family of cyclin-dependent kinase inhibitors (CKIs) which bind to and inhibit activity of cyclin/CDK (Cyclin-dependent kinase) complexes [8] and play an important role in G1 cell cycle check point progression [9]. We have recently demonstrated that loss of p21 prior to or early on in adenovirus infection allows for higher virus yields and viral mediated cytotoxity on the order of 4–5 fold [10]. Knockdown of p21 by RNA interference enhanced the replication of wild type and oncolytic adenoviruses [11]. E1A has been observed to bind to p21 in vitro, and this binding was shown to overcome a G1 arrest in the presence of overexpressed E1A [12,13]. Although E1A can be pulled down by immunoprecipitation with p21 and vice versa, a direct relationship between these proteins has not been established. The negative role of p21 in viral replication has been observed in other studies of adenovirus, human immunodeficiency virus (HIV), human cytomegalovirus (HCMV), human papilomavirus (HPV), and Minute Virus of Mice (MVM) [14,15,16,17]. It is becoming clear that p21 is an important player in the life cycle of many viruses. How exactly the p21 is altering replication and the exact mechanism of inhibition is not understood. Therefore, we evaluated many key aspects of the adenovirus life cycle to understand the impact that p21 has on viral replication.

## 2. Results

### 2.1. DNA Replication Is Significantly Increased in the Absence of p21

**Figure 1:**
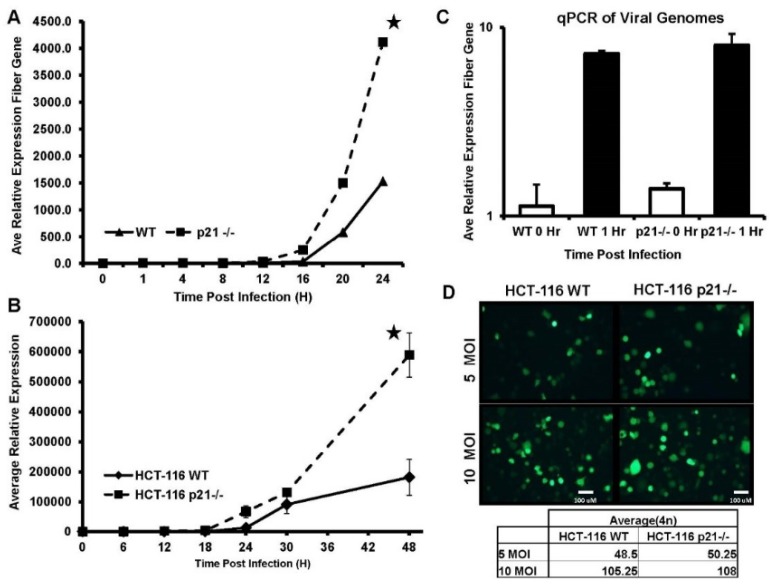
HCT-116 WT (wildtype) and p21^−/−^ cells were infected with 1 MOI (multiplicity of infection) of CN702 virus and infected cells were harvested for total DNA at indicated time points. Panel shows adenoviral DNA amplified by qPCR primers to fiber gene. Data normalized to β-Actin. Error bars S.E (**A**). HCT-116 WT and p21^−/−^ Cells were infected with 1 MOI of CN702 virus and infected cells were harvested for total DNA at indicated time points. Panel shows adenoviral DNA amplified by qPCR primers to fiber gene. Data normalized to β-Actin. Error bars represent ± S.E (**B**). Adenovirus entry by viral DNA qPCR. HCT-116 WT and p21^−/−^ cells were infected with 1 MOI of CN702 virus and infected cells were harvested for total DNA at indicated time points. Panel shows adenoviral DNA amplified by qPCR primers to fiber gene. Data normalized to β-Actin. Error bars S.E (**C**). HCT-116 WT and p21^−/−^ cells were infected with 5 or 10 MOI of CN702. At 24 h p.i. wells were imaged for GFP (green fluorescence protein) and fields were counted for GFP infected cells. No statistical difference was found in WT-infected cells vs. p21^−/−^ infected cells (**D**). Statistical significance was defined as * *p* value ≤ 0.05.

We have previously observed that there was an increase in the amount of E1A protein in adenovirus-infected p21 positive cells. However, the increased expression of E1A did not correlate to the virus output [7]. To determine whether the loss of p21 promotes a difference in either the amount or onset of viral DNA replication, HCT-116 WT and HCT-116 p21^−/−^ cells were infected with equal MOI (multiplicity of infection) of CN702 virus (ΔE3 WT Ad5) [18] and DNA was harvested at several time points. Adenoviral genome copy number was quantified by qPCR using the adenovirus fiber gene region and normalized per cell to β-Actin copy number. A time course study of viral replication in the p21 knockout and wild type HCT-116 cells at 4 h intervals revealed a significant increase in adenovirus gene copy number in p21 null HCT-116 cells (Figure 1A). This increase in viral genome amplification was not detected in HCT-116 p21 WT cells until the next four-hour interval. A time course, which included additional time points to 48 h p.i. (post infection) was included to determine whether viral DNA replication in the intact p21 wild type HCT-116 cells could reach similar levels as observed in HCT-116 p21^−/−^ cells. The 48-h time course revealed that viral DNA replication and genome accumulation in HCT-116 WT cells did not reach levels achieved in HCT-116 p21 knockout cells (Figure 1B). This increase in DNA replication is not attributed to differences in viral entry between the two cell lines as GFP fluorescence microscopy of replication-competent virus that was engineered to express GFP in viral backbone (AdTrack-HisE1A-E1B) revealed a similar amount of infected cells between the two cell lines (Figure 1C,D). These data support that viral DNA replication is susceptible to p21-mediated regulation and attenuation.

### 2.2. Oncolytic Viral DNA Replication Foci Form Earlier in p21^−/−^ Cells

**Figure 2:**
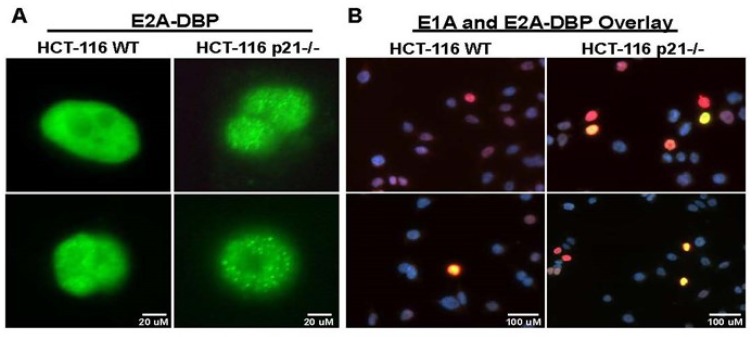

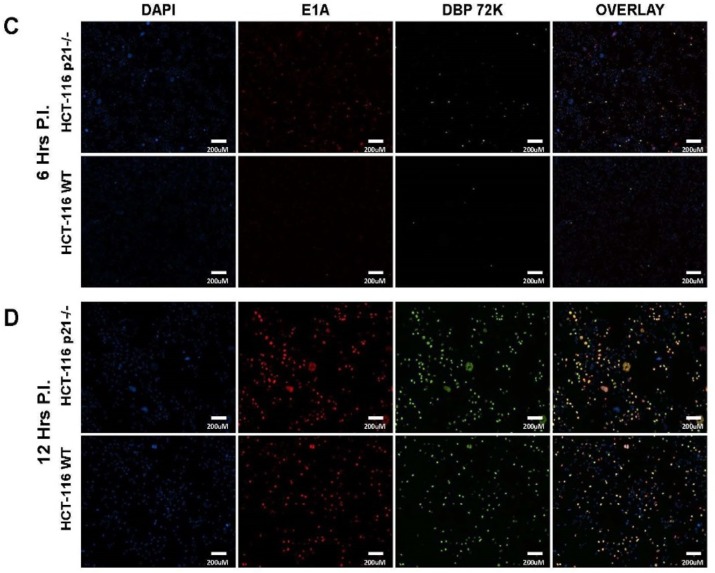
DBP foci formation in infected cells. HCT-116 WT and p21^−/−^ were plated in chamber slides and cells were infected with 5 MOI of CN702 virus. Infected cells were stopped at indicated time points and immunofluoresence microscopy was performed (**A**) 6 h p.i. 60× representatives of DBP IF at the same time point indicative of centers of viral DNA replication. 6 h p.i. 40× Field representatives of DBP (green) and E1A (red) IF at the same time point (**B**). Note in HCT-116 WT has approximately 3-fold lower number of cells that are both E1A and DBP positive for the same time point than HCT-116 p21^−/−^ cells. 6 h p.i. DBP (green) and E1A (red) IF at the same time point. Note in HCT-116 WT fewer cells are both E1A and DBP positive than HCT-116 p21^−/−^ cells (**C**). 12 h p.i. representative images. Note similar amount of dual stained cells shows no statistical significant difference in infectivity at later time point (*p* value > 0.05) (**D**).

When adenovirus replication occurs, the sites of replication within the nucleus can be visually determined by immunofluorescence microscopy of the adenovirus DNA Binding Protein (DBP) [19]. This protein binds single-stranded DNA and colocalizes to sites of replication to create foci of “replication factories” which are easily seen using immunofluorescence microscopy [20] (Figure 2A). A time course study of adenovirus replication foci formation was performed between 6 and 24 h in HCT-116 WT and HCT-116 p21^−/−^ cells infected equally with 10 MOI of replication-competent virus. Samples were stained for adenovirus DBP (green), E1A (red), and for cellular nuclei with DAPI (blue). In HCT-116 p21^−/−^ cells, DBP replication foci were observed to form as early as 4 h p.i. in infected cells (data not shown), whereas in HCT-116 WT cells, foci could not be detected until approximately 6 h p.i. (E1A and DBP double positive yellow cells) (Figure 2A,B). Analysis of the immunofluorescent microscopic images at 4× magnification further revealed a significantly higher proportion of DBP foci formation in p21 knockout cells when compared to the wild type p21 intact HCT116 cells. Similarly, E1A and DBP double positive foci (yellow) revealed much less active viral replication foci cells at 6 h p.i. in WT cells when compared to p21 knockout cells (Figure 2C). However, interestingly, at later time points (12 h p.i.), no significant difference between the double positive stained cells (yellow) was observed in wild type vs. p21^−/−^ HCT-116 infected cells. (Figure 2D). These data revealed a striking difference in early onset of viral DNA replication among p21 null cells which warranted further investigation into transcription of the viral genome.

### 2.3. Viral Transcription and Late Gene Translation Is Higher in p21^−/−^ Cells

**Figure 3:**
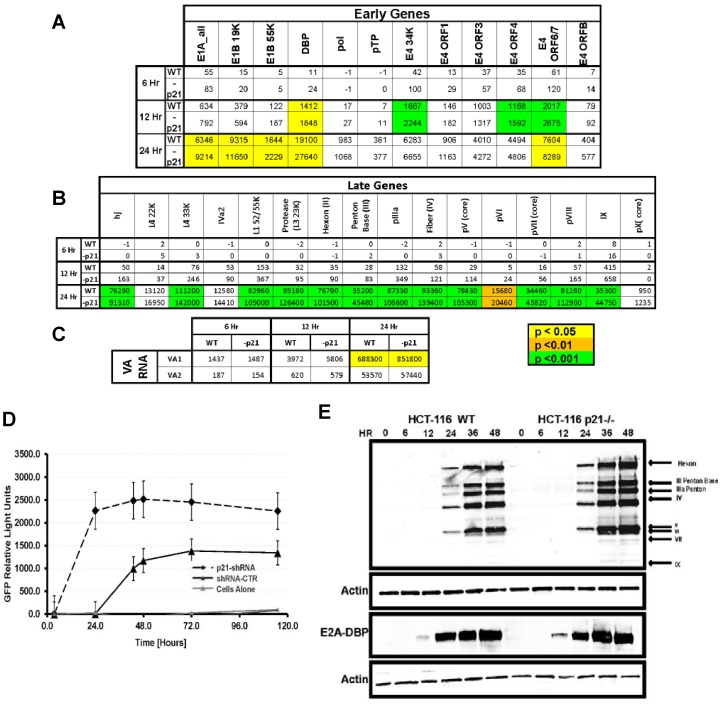
HCT-116 WT and p21^−/−^ cells were infected with 1 MOI of CN702. At indicated time points, infected cells were harvested for total RNA. 100 ng of RNA was used to run Nanostring nCounter Assay using custom Ad5 code set. Ad5 mRNA counts were normalized to internal controls and panel of housekeeping genes and quantitation of Adeno Early (**A**), Late (**B**), and VA RNA mRNA expression. The yellow, orange, and green colors indicated statistical significance with different *p* value. Significance was defined as * *p* ≤ 0.05 (**C**). HCT-116 WT cells were transfected with plasmid expressing p21 shRNA or control vector. 24 h post transfection cells were infected with 2 MOI of CN702 and 10 MOI of FFIG (Fiber-IRES-GFP) reporter virus and GFP readings were taken at indicated time points (**D**). CN702 infection time course protein expression by Westerns blot. HCT-116 WT and p21^−/−^ cells were infected with 1 MOI of CN702. At indicated time points, cells were harvested and adenovirus proteins were analyzed by Western blot (**E**).

In order to evaluate the transcriptome of the entire viral genome, a Nanostring nCounter custom code set was designed, based on the Ad5 RefSeq Genome AC_000008.1, for each gene as an alternative to multiplex qRT-PCR. Total RNA was extracted from CN702-infected HCT-116 WT and p21^−/−^ at 6, 12, and 24 h p.i. and 100 ng of RNA was used for nCounter analysis on the Ad5 code set. The data was normalized to internal controls and housekeeping genes and a *t*-test was performed for each viral gene comparing the expression in HCT-116 WT cells versus HCT-116 p21^−/−^ cells. Gene expression quantification and *p*-values for each set can be seen in Figure 3. Counts for viral genes revealed that early genes at 6 h p.i. had no statistical differences, but at 12 and 24 h p.i. there were significant differences of higher expression in E1A, E1B, E4orf6, and DBP in HCT-116 p21^−/−^ cells (Figure 3A). DNA binding protein expression by nCounter assay further confirms previous experiments that showed earlier expression by immunofluorescence microscopy in p21^−/−^ cells (Figure 2). Statistically significant differences of higher expression in most late viral genes were seen at 24 h p.i. in p21^−/−^ cells (Figure 3B,C). Interestingly, E1B55k and E4orf6 had higher expression by nCounter assay and these proteins coordinate the shuttling of viral mRNA from the cytoplasm to the nucleus [21] and may reveal a mechanism for higher amounts of late protein seen by Western blot in p21^−/−^ cells. E1A gene expression is higher in p21^−/−^ cells although its protein expression is lower compared to HCT-116 WT cells (data not shown) and may reveal differences in stability of E1A protein in p21 positive cells.

To further confirm this data, the activity of the major late promoter for CN702 virus was measured by the FFIG reporter assay [10] in HCT-116 wild type cells that were transfected with shRNA against the p21/Waf-1 or control. As shown in Figure 3D, earlier and higher GFP signals were observed in the p21^−/−^ knockout HCT116 cells compared to the wild type cells leading to the hypothesis that the expression of the structural proteins that are encoded in this region would be increased as well. A time course Western blot analysis of the late proteins revealed that there was a significant difference in the amount of late proteins expressed in p21^−/−^ compared to WT type cells at each time point (Figure 3E).

### 2.4. HCT-116 p21 WT Infected Cells Produce Smaller Plaques Which Develop Slower

**Figure 4:**
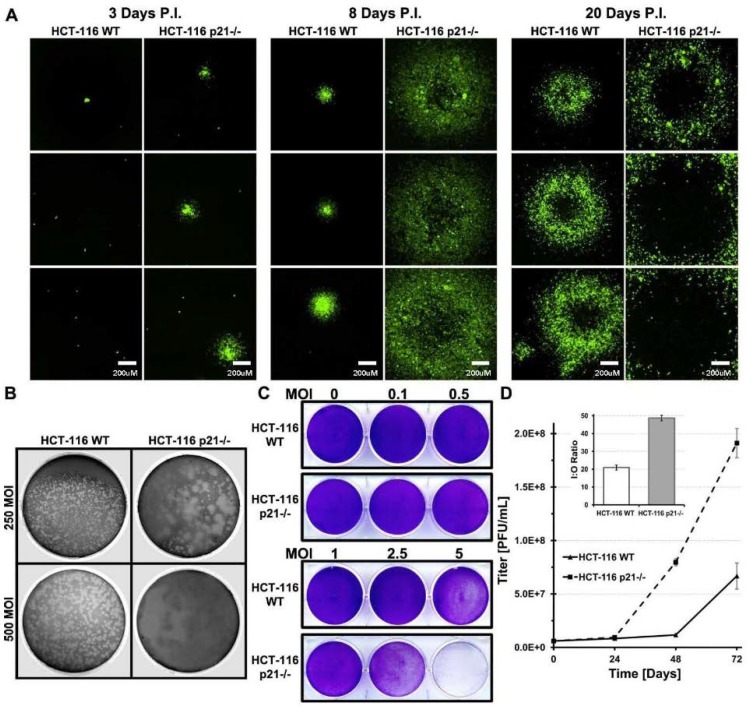
Viral burst size is significantly larger in HCT-116 p21^−/−^ cells. HCT-116 WT and p21^−/−^ cells were plated in 10-cm dishes and infected with 250 or 500 PFU of AdTrack-HisE1A-E1B virus. After 1 h, virus was removed and overlaid with 0.1% Agarose Media mixture. GFP fluorescence was imaged at indicated time points to follow viral burst (**A**). HCT-116 WT and p21^−/−^ cells were plated in 10-cm dishes and infected with 250 or 500 PFU of CN702 virus. After 1 h, virus was removed and overlaid with 0.1% Agarose Media mixture. 26 days p.i. overlays were stained with neutral red overnight (**B**). HCT-116 WT and p21^−/−^ cells were infected with various MOI of CN702. At 72 h, p.i. cell monolayers were stained with 0.5% crystal violet in methanol to visualize viral CPE (**C**). Assessing infectious particle titer over time. HCT-116 WT and p21^−/−^ cells were plated in 6 well plates and infected with 10 MOI of CN702. Cells and media were harvested at indicated time points and viral titer was done on 293 HEK cells (**D**). Statistical significance was defined as * *p* ≤ 0.05.

Plaque formation starts from a singly infected cell. At the lysis step of the replication cycle, viruses are released from the infected host cells and subsequently infect neighboring cells [22]. Plaque formation kinetics can be observed as an indirect measure of host cell lysis and spread timing. The speed at which adenovirus infection proceeds in p21^−/−^ cells compared to WT was assessed by plaque assay and visual viral burst aided by GFP expression from the adenovirus genome under the CMV promoter. Qualitative observation on the spread of virus from a single infected cell was done by GFP fluorescence microscopy over the duration of plaque development (Figure 4A). At day 1, GFP fluorescence was not visible under agar. By day 3, HCT-116 p21^−/−^ infected single cells had a visual viral burst into neighboring cells while the HCT-116 WT cells remained as single infected cells. At day 6, HCT-116 WT single cells had viral bursts similar to day 3 p21^−/−^ cells (data not shown). By day 8, HCT-116 p21^−/−^ viral bursts had grown significantly in size and necrotic centers were seen in larger viral bursts, whereas in WT cells, the viral bursts remained small and cells looked relatively healthy under light microscope conditions. In HCT-116 WT cells, viral burst can only be found under the fluorescence microscope due to the overall healthy look of the lawn of cells. At day 20, HCT-116 WT plaques were finally visible by eye but were very small. GFP fluorescence revealed small plaques compared to HCT-116 p21^−/−^ plaques. At day 25, neutral red-containing agarose was overlaid to facilitate visualization of plaques. Morphology of HCT-116 WT plaques shows the discrepancy of replication between the two cell lines (Figure 4B). Visual inspection of plaque assays in the first few days of infection revealed a difference in adenovirus cytopathic effect (CPE). HCT-116 WT cells looked healthy compared to surrounding uninfected cells and plaques could not be visually discerned until 10–12 days p.i., whereas HCT-116 p21^−/−^ plaques were visible at five days p.i. This prompted an examination of the differences in the cytopathic effect at a range of MOI in these cell lines for the same time period. Complete detachment of the cell monolayer due to CPE at a 10MOI was seen in both cell lines at four days p.i. MOI of 5 was able to produce the same removal of the cell monolayer and 1MOI was able to produce significant CPE in HCT-116 p21^−/−^ cells in the same time period, whereas in HCT-116 WT cells, the CPE effects were very mild compared to mock infected cells (Figure 4C). This data demonstrate that p21 harbors not only barriers to adenovirus replication but is protective in preventing cytopathic effects indicative of adenovirus replication.

### 2.5. Lower Amount of Oncolytic Virus in p21 Intact Cells Is Independent of Infection Time

The time point at which virus is collected for a titer comparison is typically seventy-two hours p.i. It is possible that in p21 positive cells, the delay seen in the onset of DNA replication and lower protein amounts may be overcome and the amount of packaged virus may become equal to the amount in p21^−/−^ cells if given a longer time before harvest of virus. To test this hypothesis, HCT-116 WT and p21^−/−^ cells were infected with a high MOI of virus (10 MOI) to ensure that all cells were infected and no secondary round of infection occurred during the experiments. Cells were harvested at various time points up to six days p.i. to see if the additional time allowed for the titer of HCT-116 WT cells to catch up to HCT-116 p21^−/−^ cells. As seen in Figure 4D, the titer of infectious virus in HCT-116 WT cells did not catch up to the titer of HCT-116 p21^−/−^ cells given the increased time for infection. At every time point, except for 24 h p.i., the titer of HCT-116 p21^−/−^ cells was statistically significantly higher than in p21+/+ cells. Visually, cells kept in this state appeared to be dead due to visual CPE affects and pH change in the media.

Here, we have demonstrated a role for p21 in several aspects of viral replication which, when taking into account the entire adenovirus life cycle, appears to hamper the maximum amount of virions which can be produced in a round of replication. This warrants further exploration into the mechanisms in which p21 exerts this effect, due to its role as a CDK inhibitor and regulator of the cell cycle, or a novel role for p21 in inhibiting adenovirus replication.

## 3. Materials and Methods

### 3.1. Cell Culture and Antibodies

HCT-116 WT and HCT-116 p21^−/−^ cells were maintained in McCoy’s 5A media and supplemented with 10% fetal bovine serum, gentamicin 50 µg/mL, and ciprofloxacin hydrochloride 5 µg/mL. Adenovirus antibodies used in this study consisted of mouse monoclonal antibody against E1A (M73) (Santa Cruz Biotech, Santa Cruz, CA, USA), E2A 72k DNA binding protein (B6-8) (provided as a kind gift by Dr. Arnold Levine, (Rutgers Cancer Institute of New Jersy), and rabbit polyclonal to Adenovirus type 5 capsid proteins (ab6982, Abcam, Cambridge, MA, USA). Cellular specific antibodies included mouse monoclonal against β-Actin (Sigma, St. Loius, MO, USA), mouse monoclonal anti-p21 (BD Biosciences, San Jose, CA, USA), mouse monoclonal anti-PCNA (Santa Cruz Biotech, Santa Cruz, CA, USA), chicken polyclonal anti-p21, and mouse monoclonal anti-BrdU (MoBU) (Invitrogen, Grand Island, NY, USA).

### 3.2. Indirect Immunofluorescence

Chambered slides were seeded with 2 × 10^5^ HCT-116 WT or HCT-116 p21^−/−^ cells and allowed to attach overnight. Cells were infected with 10 MOI of virus in serum-free media. One hour post infection (p.i.), virus-containing media was removed and replaced with complete media. At the indicated time points, wells were rinsed with PBS and fixed with 4% formaldehyde (methanol-free in PBS) for 15 min at room temperature. Cells were washed three times in PBS and permeabilized with 0.5% Triton X-100 in PBS for 10 min at RT (room temperature). After permeabilization, cells were washed twice and blocked for 30 min in 5% FBS in PBST (PBS with 0.05% Tween-20) then stained overnight with primary antibodies in PBST, washed three times in PBST, and incubated in secondary antibodies for 1 h at room temperature. Following secondary antibody incubation, cells were washed three times in PBST and incubated in DAPI (1 µg/mL in PBS) for 5 min. Slides were mounted in VectaShield Hardset Mounting Media (Vector Laboratories, Burlingame, CA, USA).

### 3.3. Western Blot Analysis

HCT-116 cells infected with adenovirus were washed with PBS and resuspended in ice-cold RIPA lysis buffer supplemented with protease inhibitor cocktail. The lysate was incubated on ice for 30 min then centrifuged at max speed for 10 min at 4 °C. Equal concentration of protein lysate was separated by SDS-PAGE on a 4–15% gradient gel then subsequently transferred to nitrocellulose membrane. After transfer, the membrane was blocked with Odyssey blocking buffer (LI-COR Biosciences Lincoln, NE) for 1 h at RT, then incubated in primary antibody diluted in Odyssey blocking buffer in 0.1% Tween-20 overnight at 4 °C. After washing three times in PBST, the membrane was then incubated in IRDye conjugated secondary antibody (LI-COR Biosciences, Lincoln, NE, USA) for 1 h at RT. The membrane was scanned on the Odyssey infrared imaging system.

### 3.4. Viral Stock Preparation and Real-Time PCR

Large-scale virus preparation was performed using either CsCl gradients or by commercially available adenovirus purification kit (Adenopure, Puresyn, PA, USA). The titers of the viral stocks were determined using the Adeno-XTM Rapid Titer Kit (BD Biosciences, San Jose, CA, USA). Total DNA was harvested from 1 × 10^6^ infected cells using DNeasy blood and tissue kit (Qiagen, Germantown MD, USA) according to manufacturer’s protocol. 100 ng of DNA was used as a template for quantitative real-time PCR using primer sequences corresponding to adenoviral fiber and human β-Actin gene. For reverse transcription quantitative PCR, one microgram of total RNA was reverse transcribed using QuantiTect Reverse Transcription Kit (Qiagen). Sybr green–based real-time qRT-PCR was performed using SYBR Green RT qPCR SuperMix (Invitrogen) according to the manufacturer’s instructions. All reactions were done in triplicate. Standard curves were generated by serial dilution of each sample, and the relative amount of target gene mRNA was normalized to β-Actin mRNA using the following set of primers.

Fiber SenseCCCATTGGGGGATACAAAGGGAGGAFiber AntisenseGCAGATGAAGCGCGCGCAAGACCGTE4 SenseGTAATTCACCACCTCCCGGTAE4 AntisenseGGCTCTCCACTGTCATTGTTCActin SenseGTACCACTGGCATCGTGATGGACTActin AntisenseCCGCTCATTGCCAATGGTGAT

### 3.5. Plaque Assay

Monolayers of HCT-116 WT and HCT-116 p21^−/−^ cells were grown in 10-cm plates and infected with adenovirus for 1 h in serum-free media. 1 h p.i., media containing virus was removed and an overlay of 0.5% agarose in media was added. Agarose was allowed to solidify at room temperature then plates were incubated at 37 °C. At 25 days p.i., plates were stained overnight with neutral red (50 µg/mL) in 0.5% agarose overlay.

### 3.6. Output to Input Assay

Cells were infected in six well plates at 1 MOI and harvested 72 h p.i. Cells and media went through three freeze–thaw cycles and a serial dilution of viral supernatant was titered on 293HEK cells according to Adeno-X Rapid Titer Kit protocol. The ratio of virus produced in the 72-h period (output) to initial amount of infectious virus added (input) was determined and expressed as the output to input ratio.

### 3.7. Nanostring nCounter Assay

HCT-116 WT and p21^−/−^ cells were infected with 1 MOI of CN702 virus and total RNA was extracted using TRizol according to manufacturer’s protocol (Life Technologies, Carlsbad, CA, USA). Total RNA was adjusted to 100 ng in 10 μL of DEPC (diethylpyrocarbonate) water. Samples were given to JHMI (Johns Hopkins Medicine Institute) core facility to run the nCounter analysis with thirty-eight custom probes set for the entire transcriptome of human adenovirus type 5 along with housekeeping probes for normalization. Probes sequencing for all of the thirty-eight transcripts are given in a supplementary Table S1 section.

### 3.8. Crystal Violet Staining

HCt-116 WT and HCT-116 p21^−/−^ cells were plated at 1 × 10^6^ in a 6-well plate. 24 h after plating, cells were infected with 0, 0.1, 0.5, 2.5, and 5 MOI of virus in serum-free media for 1 h. The virus was removed and replaced with complete media. The experiment was ended at 72 h post infection, the media was removed, and the cells were rinsed with phosphate-buffered saline (PBS; Invitrogen). To visualize Cytopathic effect (CPE), cells were fixed and stained with 0.5% crystal violet in 50% methanol, followed by washing with tap water to remove excess dye and thorough drying.

## 4. Discussion

Oncolytic adenoviruses are engineered to replicate and induce oncolytic cell death of cancer cells. The tumor specificity of these viruses is achieved by deleting a range of genes including E1B 55KDa or E3 from the viral backbone or by placing the viral immediate early gene E1A gene under the control of a cancer- or tissue-specific promoter and enhancer (PSE, PSA, PSMA, AFP, hTERT, etc.) that is activated in tumor cells. Despite these promising strategies, oncolytic and tissue-specific viruses have shown limited success in clinical trials. One of the main impediments of oncolytic conditionally replicating viruses were their compromised ability to replicate within the tumor environment. Our earlier studies with tissue-specific oncolytic viruses in prostate cancer patients revealed an initial viral replication (CV706) peak in the patient plasma after 3 days of viral inoculation which was depleted within ten days in the absence of preexisting circulating anti-Ad5 antibodies, suggesting a compromised viral replication [23]. Similarly, we have shown the mutual inhibitory effect of activated androgen receptor (AR) and the E1A of replicating adenovirus [24].

Overexpression of the cell-cycle-dependent kinases inhibitor, p21/Waf-1, has been shown to arrest cell cycle at the G1 or G2 phase. P21/Waf-1 is also known to interact with the proliferating cell nuclear antigen which is inversely affecting the DNA repair and replication mechanism [25]. Our previous findings suggest that incorporating an shRNA against the p21/Waf-1 in the viral backbone enhances viral oncolysis [10] where we have also shown that lower p21/Waf-1 expression induces prostate-specific promoter and enhancer (PSE/PBN) activity that was driving the expression of the E1A gene. In the current manuscript, we were specifically interested in dissecting the inhibitory mechanisms induced by the p21/Waf-1 on the oncolytic life cycle of the viruses, starting from the infection of the host cell which is mediated through the interaction between the viral capsid fiber protein and the CAR cell surface receptor proteins. To rule out any effects of higher infections in HCT116 p21^−/−^ cells because of the absence of p21/Waf-expression, which might affect the CAR receptors on these cells, we studied the GFP expression in the HCT116 p21^−/−^ and wild type cells that was engineered to expression from the viral backbone. Comparing the two cell lines revealed no significant difference between the viral entries (Figure 1C). To evaluate if there were differences between the viral DNA replication among these cell lines, we studied the viral copy number at different time point intervals using PCR and found that significant difference exists in viral replication kinetics in HCT-116 p21/Waf-1^−/−^ suggesting the role of the universal CDK inhibitor in suppressing viral DNA replication. Interestingly, we found that the total amount of viral DNA accumulated within the p21/Waf-1 knockout HCT-116 cells was at least three-fold higher when compared to the wild type HCT-116 cells. This might explain the benefits of using oncolytic viruses in cancer gene therapy as cancer cells tend to have higher proliferation and dysregulated expression of the p21/Waf-1 compared to the normal cells.

Using the mRNA expression of all of the early and late viral genes demonstrated that p21/Waf-1 suppressed both early and late gene expression while in p21/Waf-1-deficient HCT116 cells, higher mRNA expression was observed. Similarly, the VA1 and VA2, the noncoding RNA gene that is known to bind XPO5 and the miRNA processing machinery, was significantly higher in p21/Waf-1 knockout HCT-116 infected cells compared to the wild type, suggesting an overall transcriptional advantage of viral gene in p21/Waf-1 null cells. Similarly, in accordance with the mRNA expression data, we also confirmed the replication advantage of oncolytic adenovirus by plaque assay, input to output, and the FFIG reporter assays. Taken together, our data demonstrated the negative and inhibitory role of the p21/Waf-1 in the oncolytic life cycle of adenovirus.

## 5. Conclusions

We found that infection in the absence of p21 causes a significant increase in adenovirus genomes and late gene expression, which was independent of the initial viral transduction. The presence of intact p21 in host cells might be responsible for the defects in the oncolytic viral life cycle that result in the production of immature and noninfectious viral particles. These findings have translational implications in gene therapy protocols to enhance oncolytic viral replication kinetics and in developing robust viral packaging cell lines to produce high titers with lower background of noninfectious units.

## Supplementary Materials

The following are available online at http://www.mdpi.com/2072-6694/10/6/202/s1, Table S1: Generated custom probe set for human adenovirus type 5.

## References

[1] Chu R.L., Post D.E., Khuri F.R., Van Meir E.G. (2004). Use of replicating oncolytic adenoviruses in combination therapy for cancer. Clin. Cancer Res..

[2] Hermiston T.W., Kuhn I. (2002). Armed therapeutic viruses: Strategies and challenges to arming oncolytic viruses with therapeutic genes. Cancer Gene Ther..

[3] Rein D.T., Breidenbach M., Curiel D.T. (2006). Current developments in adenovirus-based cancer gene therapy. Future Oncol..

[4] Keith W.N., Bilsland A., Hardie M., Evans T.R. (2004). Drug insight: Cancer cell immortality-telomerase as a target for novel cancer gene therapies. Nat. Clin. Pract. Oncol..

[5] Rancourt C., Piche A., Gomez-Navarro J., Wang M., Alvarez R.D., Siegal G.P., Fuller G.M., Jones S.A., Curiel D.T. (1999). Interleukin-6 modulated conditionally replicative adenovirus as an antitumor/cytotoxic agent for cancer therapy. Clin. Cancer Res..

[6] Li Y., McCadden J., Ferrer F., Kruszewski M., Carducci M., Simons J., Rodriguez R. (2002). Prostate-specific expression of the diphtheria toxin a chain (dt-a): Studies of inducibility and specificity of expression of prostate-specific antigen promoter-driven dt-a adenoviral-mediated gene transfer. Cancer Res..

[7] Hoti N., Chowdhury W., Hsieh J.T., Sachs M.D., Lupold S.E., Rodriguez R. (2006). Valproic acid, a histone deacetylase inhibitor, is an antagonist for oncolytic adenoviral gene therapy. Mol. Ther..

[8] Shiyanov P., Bagchi S., Adami G., Kokontis J., Hay N., Arroyo M., Morozov A., Raychaudhuri P. (1996). P21 disrupts the interaction between CDK2 and the e2f-p130 complex. Mol. Cell. Biol..

[9] Luo Y., Hurwitz J., Massague J. (1995). Cell-cycle inhibition by independent CDK and PCNA binding domains in p21cip1. Nature.

[10] Hoti N., Chowdhury W.H., Mustafa S., Ribas J., Castanares M., Johnson T., Liu M., Lupold S.E., Rodriguez R. (2010). Armoring crads with p21/Waf-1 shrnas: The next generation of oncolytic adenoviruses. Cancer Gene Ther..

[11] Shiina M., Lacher M.D., Christian C., Korn W.M. (2009). Rna interference-mediated knockdown of p21(waf1) enhances anti-tumor cell activity of oncolytic adenoviruses. Cancer Gene Ther..

[12] Chattopadhyay D., Ghosh M.K., Mal A., Harter M.L. (2001). Inactivation of p21 by E1A leads to the induction of apoptosis in DNA-damaged cells. J. Virol..

[13] Keblusek P., Dorsman J.C., Teunisse A.F., Teunissen H., van der Eb A.J., Zantema A. (1999). The adenoviral E1A oncoproteins interfere with the growth-inhibiting effect of the CDK-inhibitor p21(CIP1/WAF1). J. Gen. Virol..

[14] Adeyemi R.O., Pintel D.J. (2012). Replication of minute virus of mice in murine cells is facilitated by virally induced depletion of p21. J. Virol..

[15] Chen Z., Knutson E., Kurosky A., Albrecht T. (2001). Degradation of p21cip1 in cells productively infected with human cytomegalovirus. J. Virol..

[16] Funk J.O., Waga S., Harry J.B., Espling E., Stillman B., Galloway D.A. (1997). Inhibition of CDK activity and PCAN-dependent DNA replication by p21 is blocked by interaction with the HPV-16 E7 oncoprotein. Genes Dev..

[17] Zhang J., Scadden D.T., Crumpacker C.S. (2007). Primitive hematopoietic cells resist HIV-1 infection via p21. J. Clin. Investig..

[18] Rodriguez R., Schuur E.R., Lim H.Y., Henderson G.A., Simons J.W., Henderson D.R. (1997). Prostate attenuated replication competent adenovirus (ARCA) CN706: A selective cytotoxic for prostate-specific antigen-positive prostate cancer cells. Cancer Res..

[19] Reich N.C., Sarnow P., Duprey E., Levine A.J. (1983). Monoclonal antibodies which recognize native and denatured forms of the adenovirus DNA-binding protein. Virology.

[20] Pombo A., Ferreira J., Bridge E., Carmo-Fonseca M. (1994). Adenovirus replication and transcription sites are spatially separated in the nucleus of infected cells. EMBO J..

[21] O’Shea C.C., Johnson L., Bagus B., Choi S., Nicholas C., Shen A., Boyle L., Pandey K., Soria C., Kunich J. (2004). Late viral RNA export, rather than p53 inactivation, determines ONYX-015 tumor selectivity. Cancer Cell.

[22] Yakimovich A., Gumpert H., Burckhardt C.J., Lutschg V.A., Jurgeit A., Sbalzarini I.F., Greber U.F. (2012). Cell-free transmission of human adenovirus by passive mass transfer in cell culture simulated in a computer model. J. Virol..

[23] DeWeese T.L., van der Poel H., Li S., Mikhak B., Drew R., Goemann M., Hamper U., DeJong R., Detorie N., Rodriguez R. (2001). A phase I trial of CV706, a replication-competent, PSA selective oncolytic adenovirus, for the treatment of locally recurrent prostate cancer following radiation therapy. Cancer Res..

[24] Hoti N., Li Y., Chen C.L., Chowdhury W.H., Johns D.C., Xia Q., Kabul A., Hsieh J.T., Berg M., Ketner G. (2007). Androgen receptor attenuation of AD5 replication: Implications for the development of conditionally replication competent adenoviruses. Mol. Ther..

[25] Cooper M.P., Balajee A.S., Bohr V.A. (1999). The C-terminal domain of p21 inhibits nucleotide excision repair in vitro and in vivo. Mol. Biol. Cell.

