# Supplementary material for: Loss of Cyclin-Dependent Kinase Inhibitor Alters Oncolytic Adenovirus Replication and Promotes More Efficient Virus Production
Source: Cancers (Basel). 2018 Jun 15;10(6):202. doi: 10.3390/cancers10060202 (PMC6025342; doi:10.3390/cancers10060202)
Supplement: Supplementary file 1 [file cancers-10-00202-s001.pdf]

Table S1. Generated custom probe set for human adenovirus type 5

Gene	Targeted Region	Target Sequence
E1A	470-570	TCATTATCACCGGAGGAATACGGGGGACCCAGATATTATGTGTTCGCTTTGCTATATGAGGACCTGTGGCATGTTTGTCTACAGTCCTGTGTCTGAACCT
E1B_19K	131-231	GGAGGATTACAAGTGGGAATTTGAAGAGCTTTTGAAATCCTGTGGTGAGCTGTTTGATTCTTTGAATCTGGGTCACCAGGCGCTTTTCCAAGAGAAGGTC
E1B_55K	741-841	GGTACGGTTTTCCTGGCCAATACCAACCTTATCCTACACGGTGTAAGCTTCTATGGGTTTAACAATACCTGTGTGGAAGCCTGGACCGATGTAAGGGTTC
IX	261-361	AGTGCAGCTTCCCGTTCATCCGCCCGCGATGACAAGTTGACGGCTCTTTTGGCACAATTGGATTCTTTGACCCGGGAACTTAATGTCGTTTCTCAGCAGC
IVa2	688-788	GAACCATTATACCGCAGTCTGGCACCCTCCGCCCGCGCTTTGTAAAAATGGCCTATGACGATCTCATCCTGGAACACAACTATGACGTTAGTGATCCCAG
pol	1323-1423	CGCATCACACGCAACTTTATGCCTCGCGCGGGAAAGATACTCTTCAACGATGTCACCTTCGCCCTGCCAAATCCGCGTTCCAAAAAGCGCACGGACTTTT
pTP	278-378	TTGCAACGACTACGTATTTGACTCGAGGGCTTACTCGCGTCTCAGGTACACCGAGCTCTCGCAGCCGGGTCACCAGACCGTTAACTGGTCCGTTATGGCC
VA1_RNA	24-124	ATAAATTCGCAAGGGTATCATGGCGGACGACCGGGGTTCGAGCCCCGTATCCGGCCGTCCGCCGTGATCCATGCGGTTACCGCCCGCGTGTCGAACCCAG
VA2_RNA	61-161	CGAGTCTCGGACCGGCCGGACTGCGGCGAACGGGGGTTTGCCTCCCCGTCATGCAAGACCCCGCTTGCAAATTCCTCCGGAAACAGGGACGAGCCCCTTT
52K	787-887	ACAAGGTGGCCGCCATCAACTATTCCATGCTTAGCCTGGGCAAGTTTTACGCCCGCAAGATATACCATACCCCTTACGTTCCCATAGACAAGGAGGTAAA
pIIIa	1178-1278	CCCTGGTTTCTACACCGGGGGATTCGAGGTGCCCGAGGGTAACGATGGATTCCTCTGGGACGACATAGACGACAGCGTGTTTTCCCCGCAACCGCAGACC

Table S1. (table continued).

Gene	Targeted Region	Target Sequence
III	362-462	CGACCTGAAAACCATCCTGCATACCAACATGCCAAATGTGAACGAGTTCATGTTTACCAATAAGTTTAAGGCGCGGGTGATGGTGTCGCGCTTGCCTACT
pVII	217-317	CCACGCCGCCACCAGTGTCCACAGTGGACGCGGCCATTCAGACCGTGGTGCGCGGAGCCCGGCGCTATGCTAAAATGAAGAGACGGCGGAGGCGCGTAGC
V	186-286	CGGGTACAGTGGAAAGGTCGACGCGTAAAACGTGTTTTGCGACCCGGCACCACCGTAGTCTTTACGCCCGGTGAGCGCTCCACCCGCACCTACAAGCGCG
pX	84-184	CTGACGGGCGGCATGCGTCGTGCGCACCACCGGCGGCGGCGCGCGTCGCACCGTCGCATGCGCGGCGGTATCCTGCCCCTCCTTATTCCACTGATCGCCG
pVI	88-188	TGAGCGGTGGCGCCTTCAGCTGGGGCTCGCTGTGGAGCGGCATTAAAAATTTCGGTTCCACCGTTAAGAACTATGGCAGCAAGGCCTGGAACAGCAGCAC
hexon	2298-2398	CTGGTACAAATGCTAGCTAACTACAACATTGGCTACCAGGGCTTCTATATCCCAGAGAGCTACAAGGACCGCATGTACTCCTTCTTTAGAAACTTCCAGC
protease	189-289	TCAAAAACATGCTACCTCTTTGAGCCCTTTGGCTTTTCTGACCAGCGACTCAAGCAGGTTTACCAGTTTGAGTACGAGTCACTCCTGCGCCGTAGCGCCA
DBP	1049-1149	TTCCGGCAAGTCTTGCGGCATGTTCTTCTCTGAAGGCGCAAAGGCTCAGGTGGCTTTTAAGCAGATCAAGGCTTTTATGCAGGCGCTGTATCCTAACGCC
100K	1521-1621	TCAGGAATCTTGCCCGCCACCTGCTGTGCACTTCCTAGCGACTTTGTGCCCATTAAGTACCGCGAATGCCCTCCGCCGCTTTGGGGCCACTGCTACCTTC
33K	460-560	CCAACGAACCCGTATCGACCCGCGAGCTTAGAAACAGGATTTTTCCCACTCTGTATGCTATATTTCAACAGAGCAGGGGCCAAGAACAAGAGCTGAAAAT
22K	367-467	GCTACCGCTCATGGCGCGGGCACAAGAACGCCATAGTTGCTTGCTTGCAAGACTGTGGGGGCAACATCTCCTTCGCCCGCCGCTTTCTTCTCTACCATCA

Table S1. (table continued).

Gene	Targeted Region	Target Sequence
pVIII	112-212	CCCACATGATATCCCGGGTCAACGGAATCCGCGCCCACCGAAACCGAATTCTCTTGGAACAGGCGGCTATTACCACCACACCTCGTAATAACCTTAATCC
E3_12.5K	39-139	CTGGTCCACTGTCGCCGCCACAAGTGCTTTGCCCGCGACTCCGGTGAGTTTTGCTACTTTGAATTGCCCGAGGATCATATCGAGGGCCCGGCGCACGGCG
E3_CR1-alpha0	85-185	TCTTTATTCTTATACTAACGCTTCTCTGCCTAAGGCTCGCCGCCTGCTGTGTGCACATTTGCATTTATTGTCAGCTTTTTAAACGCTGGGGTCGCCACCC
E3_gp19K	0-100	ATGATTAGGTACATAATCCTAGGTTTACTCACCCTTGCGTCAGCCCACGGTACCACCCAAAAGGTGGATTTTAAGGAGCCAGCCTGTAATGTTACATTCG
10.5_kD	23-123	CGCCGCTACCGGACTTACATCTACCACAAATACACCCCAAGTTTCTGCCTTTGTCAATAACTGGGATAACTTGGGCATGTGGTGGTTCTCCATAGCGCTT
E3_RID-alpha	158-258	CACTGTGGTCATCGCCTTTATCCAGTGCATTGACTGGGTCTGTGTGCGCTTTGCATATCTCAGACACCATCCCCAGTACAGGGACAGGACTATAGCTGAG
E3_RID-beta	145-245	CCGAAGCCTGGTTATATGCAATCATCTCTGTTATGGTGTTCTGCAGTACCATCTTAGCCCTAGCTATATATCCCTACCTTGACATTGGCTGGAAACGAAT
E3_14.7K	87-187	CGCATGAATCAAGAGCTCCAAGACATGGTTAACTTGCACCAGTGCAAAAGGGGTATCTTTTGTCTGGTAAAGCAGGCCAAAGTCACCTACGACAGTAATA
U_exon	5-105	GATAGTGGGTGCGGATGGACAGGAACAGGAGGAAACTGACATTCCATTTAGATTGTGGAGAAAGTTTGCAGCCAGGAGGAAGCTGCAATACCAGAGCTGG
fiber	592-692	CACAAAATGGAAAACTAGGACTAAAGTACGGGGCTCCTTTGCATGTAACAGACGACCTAAACACTTTGACCGTAGCAACTGGTCCAGGTGTGACTATTAA
E4_ORF6_7	321-421	GTGTGGAATATAACACCTAAGAATATGTCTGTTACCCATGATATGATGCTTTTTAAGGCCAGCCGGGGAGAAAGGACTGTGTACTCTGTGTGTTGGGAGG
ACTB	1062-1162	ATCAAGATCATTGCTCCTCCTGAGCGCAAGTACTCCGTGTGGATCGGCGGCTCCATCCTGGCCTCGCTGTCCACCTTCCAGCAGATGTGGATCAGCAAGC
CLTC	290-390	GGGTATCAACCCAGCAAACATTGGCTTCAGTACCCTGACTATGGAGTCTGACAAATTCATCTGCATTAGAGAAAAAGTAGGAGAGCAGGCCCAGGTGGTA
HPRT1	354-454	TAGCCCTCTGTGTGCTCAAGGGGGGCTATAAATTCTTTGCTGACCTGCTGGATTACATCAAAGCACTGAATAGAAATAGTGATAGATCCATTCCTATGAC
